# CL-705G: a novel chemical Kir6.2-specific K_ATP_ channel opener

**DOI:** 10.3389/fphar.2023.1197257

**Published:** 2023-06-20

**Authors:** Ivan Gando, Manuel Becerra Flores, I.-Shan Chen, Hua-Qian Yang, Tomoe Y. Nakamura, Timothy J. Cardozo, William A. Coetzee

**Affiliations:** ^1^ Biochemistry and Molecular Pharmacology, NYU Grossman School of Medicine, New York, NY, United States; ^2^ Phamacology, Wakayama Medical University, Wakayama, Japan; ^3^ Cyrus Tang Hematology Center, Soochow University, Suzhou, China

**Keywords:** KATP channels, kir6.2, potassium channel opener, structure-activity relationship (SAR), cardioprotection

## Abstract

**Background:** K_ATP_ channels have diverse roles, including regulation of insulin secretion and blood flow, and protection against biological stress responses and are excellent therapeutic targets. Different subclasses of K_ATP_ channels exist in various tissue types due to the unique assemblies of specific pore-forming (Kir6.x) and accessory (SURx) subunits. The majority of pharmacological openers and blockers act by binding to SURx and are poorly selective against the various K_ATP_ channel subclasses.

**Methods and Results:** We used 3D models of the Kir6.2/SUR homotetramers based on existing cryo-EM structures of channels in both the open and closed states to identify a potential agonist binding pocket in a functionally critical area of the channel. Computational docking screens of this pocket with the Chembridge Core chemical library of 492,000 drug-like compounds yielded 15 top-ranked “hits”, which were tested for activity against K_ATP_ channels using patch clamping and thallium (Tl^+^) flux assays with a Kir6.2/SUR2A HEK-293 stable cell line. Several of the compounds increased Tl^+^ fluxes. One of them (CL-705G) opened Kir6.2/SUR2A channels with a similar potency as pinacidil (EC_50_ of 9 µM and 11 μM, respectively). Remarkably, compound CL-705G had no or minimal effects on other Kir channels, including Kir6.1/SUR2B, Kir2.1, or Kir3.1/Kir3.4 channels, or Na^+^ currents of TE671 medulloblastoma cells. CL-705G activated Kir6.2Δ36 in the presence of SUR2A, but not when expressed by itself. CL-705G activated Kir6.2/SUR2A channels even after PIP_2_ depletion. The compound has cardioprotective effects in a cellular model of pharmacological preconditioning. It also partially rescued activity of the gating-defective Kir6.2-R301C mutant that is associated with congenital hyperinsulinism.

**Conclusion:** CL-705G is a new Kir6.2 opener with little cross-reactivity with other channels tested, including the structurally similar Kir6.1. This, to our knowledge, is the first Kir-specific channel opener.

## Introduction

The opening of ATP-sensitive K^+^ (K_ATP_) channels at the sarcolemmal/plasmalemmal membrane is regulated by alterations in intracellular ATP and ADP levels (Lederer and Nichols, 1989). As such, K_ATP_ channels couple intracellular energy metabolism to electrical excitability. In glucose-sensitive neurons in the hypothalamus, for example, K_ATP_ channels link low glucose levels to feeding behavior (Miki et al., 2001). In pancreatic β-cells, K_ATP_ channel closure in response to increased plasma glucose and intracellular ATP/ADP ratio triggers insulin secretion (Tarasov et al., 2006). In vascular smooth muscle, the vascular endothelium, and in pericytes, K_ATP_ channels couple the tissue energy demand to vasodilatation and to an increased blood flow (Tinker et al., 2018; Zhao et al., 2020; Sancho et al., 2022). In the heart, K_ATP_ channels are activated during rapid heart rates to promote adaptive shortening of the cardiac action potential (Zingman et al., 2011; Foster and Coetzee, 2016), and their opening (and surface trafficking) have a key role on the protective effects of ischemic preconditioning (Yang et al., 2016). Genetic variations in K_ATP_ channel genes (channelopathies) that affect channel opening or trafficking are associated with human disorders, including congenital hyperinsulinism, neonatal diabetes, Brugada syndrome, and Cantú syndrome (McClenaghan and Nichols, 2022). Pharmacologically targeting K_ATP_ channels represents a rich opportunity for therapeutic intervention to influence a range of physiological and pathophysiological processes.

At the molecular level, a K_ATP_ channel is composed of two types of transmembrane proteins (Foster and Coetzee, 2016; Tinker et al., 2018; Yang et al., 2022). The pore-forming component of the channel is a tetramer of inward rectifier potassium channel (Kir6) subunits. The Kir6 subfamily has two members, Kir6.1 and Kir6.2, respectively products of the *KCNJ8* and *KCNJ11* genes. Each Kir6. x subunit has an intracellular N-terminus, two transmembrane helixes, a pore-forming consensus sequence (Gly-Phe-Gly) and an intracellular C-terminus. Intracellular ATP reduces gating by binding to a nucleotide binding pocket composed by N- and C-termini of adjacent Kir6. x subunits. This nucleotide binding pocket partially overlaps with a binding site for phosphoinositol (4,5) bisphosphate (PIP_2_), which is a powerful stimulus for K_ATP_ channel opening. In addition to the pore-forming component, the K_ATP_ channel has four regulatory subunits named sulphonylurea receptors (SUR). There are two such regulatory subunits, SUR1 and SUR2, respectively encoded by the *ABCC8* and *ABCC9* genes. Both SURx subunits are alternatively spliced, but the most commonly studied are two of the SUR2 splice variants, SUR2A and SUR2B, which differ from each other in the distal 42 amino acids of the C-terminus as a result of alternative splicing events. The SURx subunits are members of the ATP-binding cassette (ABC) superfamily of proteins, have an extracellular N-terminus, a transmembrane domain 0 (TMD0) that consists of five transmembrane regions, and TMD1 and TMD2; each consisting of six membrane helices. An intracellular nucleotide binding fold (NBF1) is present between TMD1 and TMD2, and another (NBF2) is present in the intracellular C-terminus. Nucleotide binding (particularly ADP) to these NBFs stimulates K_ATP_ channel opening.

There are several subtypes of K_ATP_ channels, often with distinct tissue distributions (Foster and Coetzee, 2016). Kir6.2 in combination with SUR2A, for example, is considered as the molecular component of the cardiac ventricular K_ATP_ channel. K_ATP_ channels in the β-cells of the islets of Langerhans in the pancreas are composed of Kir6.2/SUR1 subunits, whereas vascular smooth muscle channels are composed of Kir6.1/SUR2B subunits. There are dozens of compounds that can block or activate K_ATP_ channel activity (Tinker et al., 2018). A therapeutic challenge is that most of these compounds lack subclass specificity, leading to significant cross-reactivity of K_ATP_ channel openers and blockers amongst the various K_ATP_ channel subtypes. Here, we describe a novel compound that binds to Kir6.2 to activate K_ATP_ channels, without affecting other members of the inward rectifying K^+^ channel subfamily. To our knowledge, this is the first Kir-specific channel opener.

## Materials and methods

### cDNA constructs and mutagenesis

K_ATP_ channel subunit constructs used are Kir6.2-myc, Flag-SUR1, Flag-SUR2A, and Kir6.2Δ36. Kir6.2-F55L, Kir6.2-T62M, Kir6.2-R301C, SUR1-V715G, SUR1-S1386P, and SUR1-L1390P were provided by Dr. Show-Ling Shyng (Oregon Health and Science University, Portland, United States).

### Cell culture and transfection

HEK-293 cells were cultured in DMEM with 10% fetal bovine serum; 100 U/mL penicillin; 100 μg/mL streptomycin at 37°C in a humidified 5% CO_2_ atmosphere. Kir6.2/SUR2A and Kir6.1/SUR2B stable HEK-293 cell lines were from Dr. A. Tinker (University of London, United Kingdom). For transient transfections, cells were plated in a 60 mm dish and cultured for 24 h. The next day cells were co-transfected with 0.2 µg of Kir6.2-myc, Kir6.2 Δ36, or mutant Kir6.2 with 1.8 µg of Flag-SUR1 or Flag-SUR2A utilizing Lipofectamine 2000 (Thermo Fisher Scientific). Twenty-4 h after transfection, cells were re-plated onto glass coverslips or 96-well plates for experimentation.

### Thallium flux assay

The cells were plated at a density of 3.0–4.0 × 10^5^ cells/well in black, flat-bottom, 96-well plates and cultured overnight until confluent. The cells were then incubated for 1 h with FluxOR II reagent dye in loading buffer, followed by washing and 10 min of exposure to 80 µL of assay buffer containing DMSO (vehicle control), pinacidil (K_ATP_ channel activation control), the K_ATP_ channel inhibitor glibenclamide or test compounds. All compounds were dissolved in DMSO. Fluorescence was recorded at excitation/emission wavelengths of 495 nm/510 nm using a microplate reader equipped with a dispenser unit for adding compounds during the experiment (FlexStation 3, Molecular Devices). Fluorescence intensities were recorded every 2 s for the duration of the experiment (∼2 min). An averaged baseline fluorescence intensity value was calculated from at least 8 repeated measurements, after which a stimulus buffer containing Tl_2_SO_4_ (20 µL) was added to each well. The fluorescence intensity of each well was normalized with an average baseline fluorescence intensity value from 8 repeated measurements prior to stimulus buffer injection. Given that the Tl^+^ flux assay is cumulative, the maximum fluorescence attained during a prolonged experiment have low sensitivity and reproducibility. By contrast, the initial rate is a more sensitive index of activity, as also used by others (Carmosino et al., 2013). Moreover, the initial rate of Tl^+^ flux is more comparable to data obtained with patch clamping (Du et al., 2015; Philippaert et al., 2018). For this reason, we used the initial rate of Tl^+^ flux as an index of channel activity.

### Patch clamp electrophysiology

Cells, plated on coverslips, were subjected to patch clamping using a Multiclamp 700 A amplifier (Molecular Devices, San Jose, CA, United States). Borosilicate glass electrodes were pulled with a DMZ Universal Puller (Zeitz, Germany). For inside-out K_ATP_ current recordings, pipettes (3–5 MΩ resistance) were filled with a pipette solution containing (in mM): 110 K-gluconate, 30 KCl, 2 CaCl_2_, 1 MgCl_2_, 10 HEPES, at pH 7.4. The bath solution contained (in mM): 110 K-gluconate, 30 KCl, 1 EGTA, 1 MgCl_2_, 10 HEPES, at pH 7.2. Following patch excision, the pipette potential was held at +80 mV, which corresponds to a membrane potential of −80 mV. Currents were recorded immediately after patch excision. Recordings with significant rundown were discarded. Compounds were applied using a rapid solution changer (RSC160, BioLogic SAS, Seyssinet-Pariset, France). For whole-cell K_ATP_ current recordings, the pipettes (2∼3 MΩ) were filled with (in mM): 110 K-gluconate, 10 KCl, 5 NaCl, 1 MgCl_2_, 5 EGTA, 10 HEPES, at pH 7.2. The bath solution contained (in mM): 110 K-gluconate, 10 KCl, 2 CaCl_2_, 1 MgCl_2_, 10 HEPES, and 10 glucose at pH 7.4. Whole-cell current–voltage relationships were obtained using a 2 s ramp protocol from 0 mV to −80 mV and back to 0 mV. Compound application was performed using the rapid solution changer. Data were filtered at 1 kHz (8-pole Bessel response), digitized (Digidata 1550A; Molecular Devices), acquired at 10 kHz and analyzed using pClamp 10 (Molecular Devices).

For Kir2.1 current recordings, the pipettes (2∼3 MΩ) were filled with (in mM): 110 mM KCl, 5 NaCl, 1 MgCl_2_, 5 EGTA, 10 HEPES at pH 7.2. The bath solution contained (in mM): 140 NaCl, 4 KCl, 1 MgCl_2_, 10 HEPES, 2 CaCl_2_ at pH 7.4. The Kir2.1 current was recorded from a holding potential of 0 mV using 200 ms voltage steps −140 to +40 mV in 10 mV increments. Currents were normalized by cell size and reported as current density (pA/pF).

Voltage-gated sodium currents were recorded using a QPatch II (Sophion Bioscience, Denmark) with 48 channel QPlates, with a one patch hole per channel. The QPlates had a hole diameter of ∼1 μm and a resistance of ∼2 MΩ. The QPlates were primed with an internal solution containing (in mM): 110 CsF, 10 CsCl, 10 NaCl, 10 EGTA, 10 HEPES at pH 7.2 (osmolarity ∼286 mOsm). TE671 cells were washed and re-suspended with extracellular solution containing (in mM): 140 NaCl 4 KCl, 2 CaCl_2_, 1 MgCl_2_, 5 glucose, 10 HEPES at pH 7.4 (osmolarity ∼298 mOsm) to ensure complete removal of media before being loaded into the QPatch II. Whole-cell configuration was achieved by the QPatch II utilizing a GΩ seal formation and rupture protocol optimized for TE671 cells (the protocol is available upon request). After establishing a successful whole-cell configuration, cells were held at −120 mV and a 3 ms voltage step to 0 mV was repetitively applied at a rate of 0.03 Hz for 2 min to assess the stability of the recordings. Current-voltage relationships were measured from a holding potential of −120 mV, the membrane was depolarized to voltages between −80 mV and 60 mV for 300 ms. Series resistances were compensated by ∼85%. Currents were low-pass filtered at 10 kHz with an 8-pole Bessel filter response and recorded at a sampling rate of 25 kHz. Currents were normalized to cell size and reported as current density (pA/pF).

### Oocyte two-electrode voltage-clamping

Oocytes were isolated from female *Xenopus laevis* as previously described (Chen et al., 2022). Frogs were purchased from Watanabe Zoushoku (Hyogo, Japan) and housed at 18°C in the animal facility of Wakayama Medical University. After anesthetization of the frog with 0.15% tricaine (Tokyo Chemical Industry, Tokyo, Japan) through skin absorption for 30 min, oocytes were dissociated on ice after making a small abdominal incision. Follicle cells were removed with 2 mg mL^−1^ collagenase type I (Sigma-Aldrich, St. Louis, MO, Unites States) for 6 h in a solution containing (in mM) 88 NaCl, 1 KCl, 2.4 NaHCO_3_, 0.3 Ca(NO_3_)_2_, 0.41 CaCl_2_, 0.82 MgSO_4_ and 15 HEPES at pH 7.6. The cRNA of mouse Kir3.1 and rat Kir3.4 were transcribed with the mMessage mMachine T3 Transcription Kit (Invitrogen, Carlsbad, CA, Unites States) from their linearized cDNA in *pBluescript II* and then were injected into each oocyte (25 ng each for Kir3.1 and Kir3.4). After 3–5 days of the injection, oocytes were used for two-electrode voltage-clamp recordings.

GIRK currents were recorded from oocytes at room temperature using pClamp 10 (Molecular Devices), a two-electrode voltage clamp amplifier OC-725C (Warner Instruments, Holliston, MA, Unites States) and an analog-to-digital converter Digidata 1550 B (Molecular Devices). Recoding electrodes (0.2–0.5 MΩ) were filled with a pipette solution containing 3 MK-acetate and 10 mM KCl. CL-705G was applied to an extracellular high K^+^ solution that contained (in mM): 96 KCl, 3 MgCl_2_, 10 HEPES, at pH 7.3 adjusted with *n*-methyl-d-glucamine. The background leakage current was determined by replacing the extracellular K^+^ with the impermeable Na^+^ and data with significant leak were excluded from the analysis.

### Hypoxia-reoxygenation with mouse cardiomyocytes

Mouse ventricular myocytes were isolated using a Langendorff-free method (Ackers-Johnson et al., 2016). Following calcium reintroduction, cardiomyocytes were resuspended in culture medium (M199, 0.1% BSA, CD Lipid, Insulin-Transferrin-Selenium) and plated in 96-well plates coated with 1% Geltrex in M199 media. Cells were allowed to attach in an O_2_/CO_2_ incubator containing a humidified atmosphere of 5% CO_2_ at 37°C for 2 h. Cells were then treated with phenylephrine (5 µM) in the presence or absence of glibenclamide (1 µM), or with CL-705G (100 µM) for 24 h. Hypoxia was induced by placing cells in an oxygen controlled chamber (Biospherix, Lacona, NY) containing 1% O_2_, 5% CO, and 94% N_2_ at 37°C for 2 h. Cells were reoxygenated by changing media and placing them in normoxic incubator for 18 h. At the end of the sequence of hypoxia/reoxygenation, cardiomyocyte survival was measured by determining total cellular ATP content (CellTiter Glo 2.0 Cell Viability assay, Promega).

### Molecular modeling, docking, and virtual library screening

All molecular modeling, alignments and computational simulations were performed with the Internal Coordinates Mechanics software, (ICM-Pro, Molsoft LLC, La Jolla CA). For the open conformation K_ATP_ channel, two 3D models were evaluated, the 3.7 Å, near-atomic resolution cryo-EM structure of Kir6.2 in an open conformation (PDB 7S5X) and, for atomic resolution, a homology model of Kir6.2 in the conformation observed in the PIP_2_-bound, presumably open conformation, of Kir3.2 (PDB: 3SYQ; 3.44 Å). To generate the latter, zero-end gap global sequence alignment between the human Kir6.2 sequence (Uniprot: Q14654) and the Kir3.2 sequence produced a *p*-value of 10^−32^ for 3D structural similarity. In prior work, precise homology modeling produces 3D structural models of equivalent accuracy to the experimental template structure (3.44 Å) when the *p*-value is so low (Abagyan and Batalov, 1997). A homology model of the human Kir6.2 channel was built as previously described (Cardozo et al., 1995), by using 3SYQ as a structural template. For the closed conformation of K_ATP_ channel, we used PDB: 6C3P, which was resolved by cryo-EM at 5.6 Å, as the starting point for a 3D model. For both 7S5X and 6C3P, missing side-chains were built into the model, followed by Biased Probability Monte Carlo energy minimization in order to optimize the energy of the structure and correct any modeling errors from the density (Abagyan and Totrov, 1994). For point mutations, individual models exhibiting palmitoylation or mutations of cysteine-166 (Cys^166^) were generated using the “modify” command in ICM-Pro, which replaces, in the open conformation homology model or the closed conformation refined model, a specified side chain atom group and bonding pattern (in this case Cys^166^) with the side chain atoms and bonding pattern of a different specified amino acid. The newly introduced side-chain, neighboring sidechains and the local backbone are then all subjected to Monte Carlo energy minimization as a single ensemble. Docking was performed with three orthogonal programs: ICM-Pro, AutoDock (Morris et al., 2008) and CB-Dock2 (Liu et al., 2022). Virtual library screening was performed with ICM-Pro: briefly, grid potentials for van der Waals and electrostatics were generated from the Kir6.2 model coordinates for the region of the pocket in the structures and full-atom models of the compounds were docked into these grids. The Chembridge Core chemical library (Chembridge Corp. La Jolla CA) was screened as mol format files for each compound, and the CL-705G compound structure is encoded as a mol formatted data structure within this library. Compounds were considered hits with an ICM energy score of < −32. The compounds in Table 1 are all the compounds achieving this docking score without common substructures.

**TABLE 1:** Properties of compounds.

ChemBridge Id	Name	Compound structure
68323670	N-(2-hydroxyethyl)-1-[1-(3-phenylbutyl)-4-piperidinyl]-1H-1,2,3-triazole-4-carboxamide	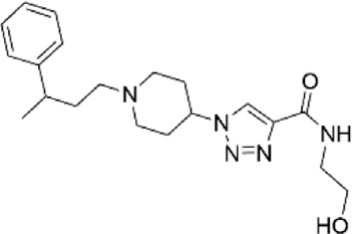
44516625	N∼3∼-{[1-(4-biphenylylmethyl)-3-oxo-2-piperazinyl]acetyl}-beta-alaninamide	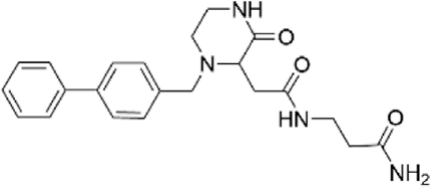
68348812	N-[2-(2,4-dimethylphenoxy)ethyl]-3-(2,5-dioxoimidazolidin-4-yl)-N-methylpropanamide	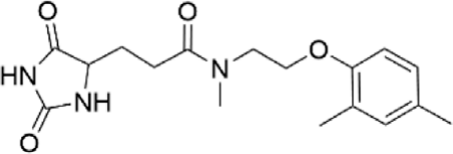
67698044	3-(2,5-dioxo-4-imidazolidinyl)-N-[(5-fluoro-1H-indol-2-yl)methyl]-N-methylpropanamide	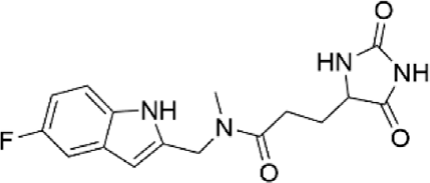
73948788	N-[(5-chloro-1H-benzimidazol-2-yl)methyl]-3-(2,5-dioxo-4-imidazolidinyl)-N-methylpropanamide	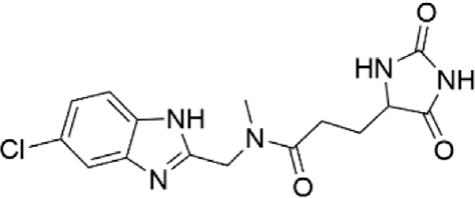
12533394	(3S,9aR)-8-(3-fluoro-4-methylbenzyl)-3-(1H-imidazol-4-ylmethyl)tetrahydro-2H-pyrazino [1,2-a]pyrazine-1.4(3H,6H)-dione	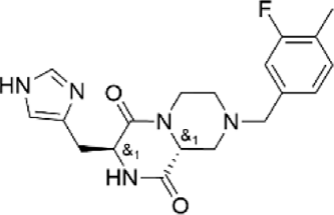
99756088	N-{(3R*,4R*)-1-[(5-tert-butyl-1H-pyrazol-3-yl)methyl]-3-hydroxypiperidin-4-yl}nicotinamide	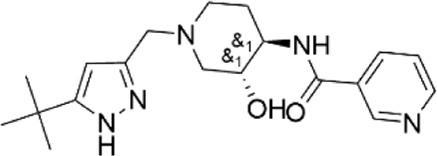
69769323	methyl 3-(benzoylamino)-6-({methyl [1-(4-methyl-1,3-thiazol-2-yl)ethyl]amino}methyl)thieno [2,3-b]pyridine-2-carboxylate	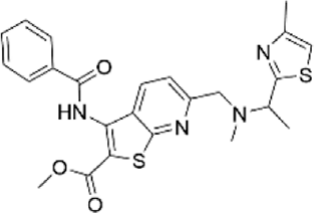
41380967	N-{(3R*,4R*)-3-hydroxy-1-[(5-methyl-2-thienyl)methyl]piperidin-4-yl}nicotinamide	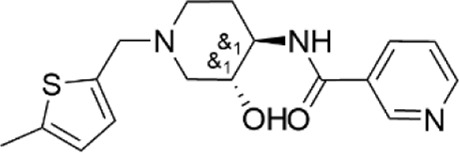
85899912	N-[(3R*,4R*)-1-(3-cyano-4,6-dimethylpyridin-2-yl)-3-hydroxypiperidin-4-yl]pyrazine-2-carboxamide	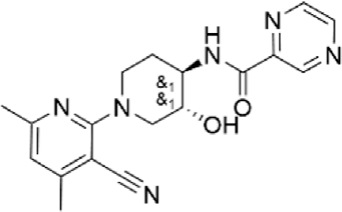
78673048	(3S*,9aR*)-8-{[1-(4-fluorophenyl)-1H-pyrazol-4-yl]methyl}-3-(1H-imidazol-4-ylmethyl)tetrahydro-2H-pyrazino [1,2-a]pyrazine-1.4(3H,6H)-dione	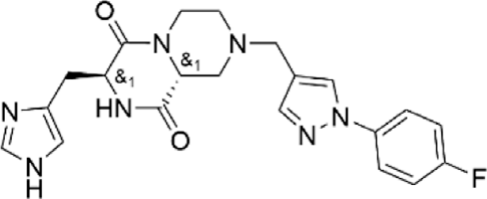
23526545	(3S,9aR)-3-(4-aminobutyl)-8-(2-naphthoyl)tetrahydro-2H-pyrazino [1,2-a]pyrazine-1.4(3H,6H)-dione	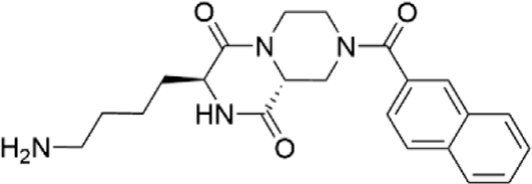
33585536	5-({3-[4-(2-methoxyphenyl)-1-piperazinyl]-1-piperidinyl}carbonyl)-1,2-dihydro-3H-pyrazol-3-one	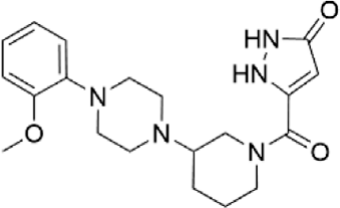
12807730	5-{[(3S*,4S*)-3-hydroxy-4-(2-naphthyl)piperidin-1-yl]carbonyl}pyrimidine-2.4(1H,3H)-dione	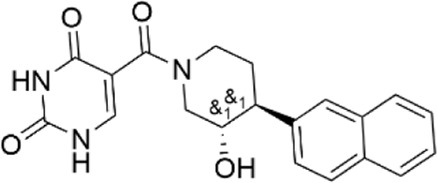
70862985 (CL-705G)	5-[(3-{2-[2-(trifluoromethyl)phenyl]ethyl}-1-piperidinyl)carbonyl]-1,2-dihydro-3H-pyrazol-3-one	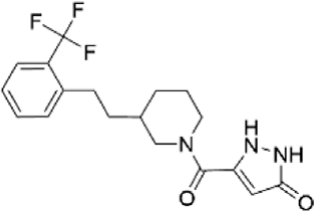

Additional information of each compound is available at Hit2Lead (https://www.hit2lead.com).

### Statistics

When comparing two groups, we used the Student’s t-test. A one-way or two-way ANOVA was used for comparison of multiple groups, followed by the Dunnett’s *t*-test for comparisons to a single control. A value of *p* < 0.05 was considered significant. The statistical model underlying the homology modeling alignments has previously been described (Abagyan and Batalov, 1997).

### Data availability statement

All relevant material is contained within the article or the Supplementary Material.

## Results

### Identification of a potential druggable pocket in Kir6.2

**FIGURE 1:**
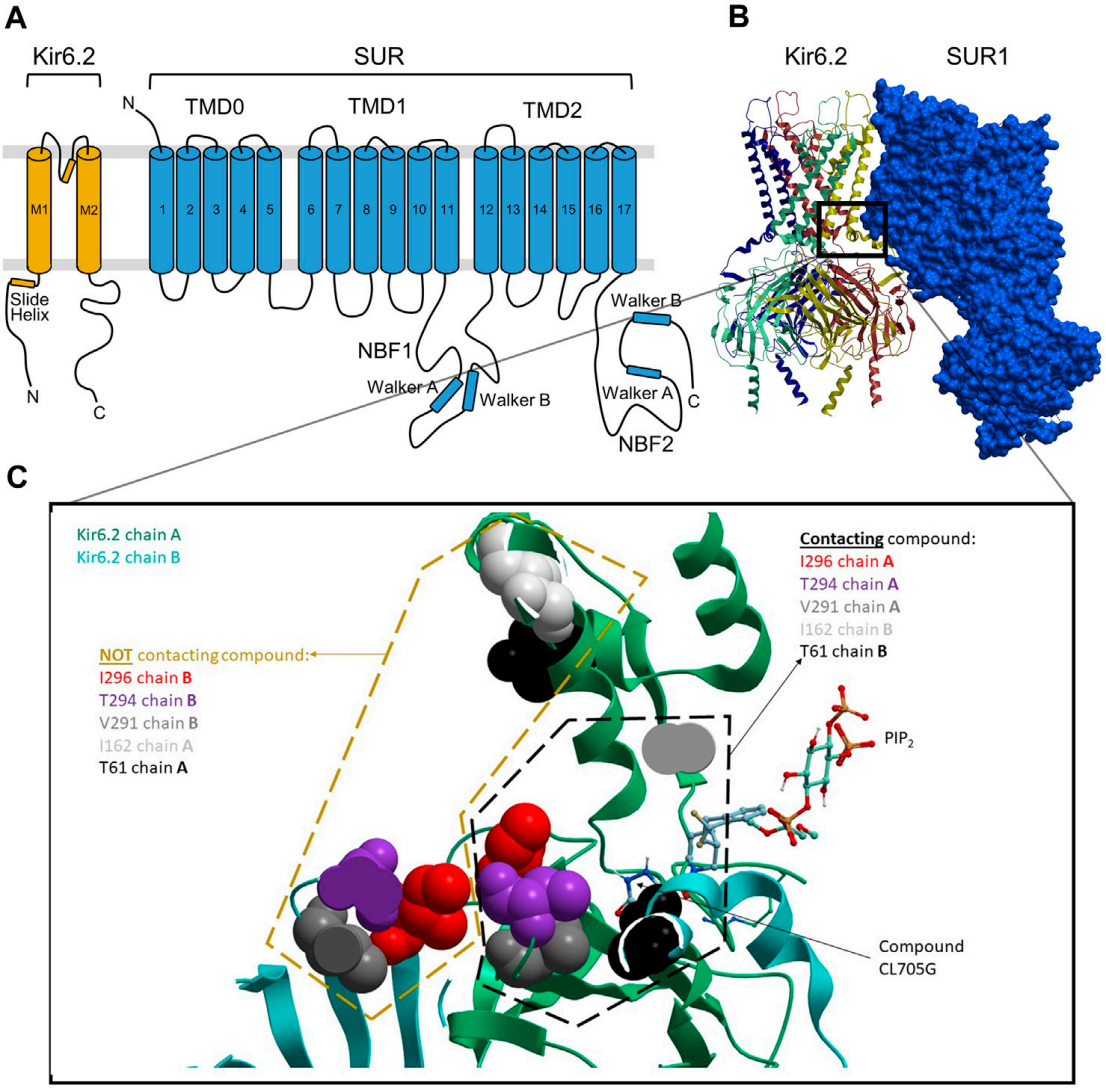
Docked location of compound CL-705G in Kir6.2. **(A)** Topology of Kir6.2 and SURx with some of the major structural domains and elements labeled. **(B)** Cryo-EM-derived, 3D structural model of Kir6.2/SUR1 (PDB: 6JB1), with the four Kir6.2 monomers depicted in distinct colors. **(C)** The boxed area in panel b is expanded to zoom in on the putative CL-705G binding pocket, which is formed by adjacent Kir6.2 transmembrane (TM) helices, as well as loop 0 and the TM1 helix of SUR1 TMD0. The larger pocket in which this compound docks is also predicted to be occupied by PIP_2,_ as depicted.

There are now several cryo-EM structures available for Kir6.2/SUR1 and Kir6.1/SUR2B channels. Currently, most are in the closed state (e.g., 6C3P) (Lee et al., 2017), but an atomic-resolution X-ray crystallographic structure of mouse PIP_2_-bound Kir3.2 (PDB: 3SYQ, 3.44 Å) (Whorton and MacKinnon, 2011), a few high-resolution X-ray structures of fowl Kir2.2 in open and closed conformations (e.g., PDBs: 6M84, 2.81 Å; 5KUK 2 Å) (Lee et al., 2016; Zangerl-Plessl et al., 2020), and a recent near-atomic-resolution ensemble of cryo-EM structures of open Kir6.2/SUR1 (e.g., PDB 7S5V, 7S5X) are available. We used our previously published methods (Martinez-Ortiz and Cardozo, 2018) to refine these existing structures and to generate maximally realistic 3D models of the Kir6.2/SUR homotetramers in both the open and closed states (Yang et al., 2020). We previously found that S-palmitoylation of a specific Cys-166 residue in the M2 region of Kir6.2 promotes K_ATP_ channel opening by allosterically connecting M2 to the PIP_2_ binding site (Yang et al., 2020). The cavity occupied by the palmitoyl moiety and PIP_2_ in this model represents a potentially druggable pocket influencing channel opening (Figure 1).

### Virtual library screening to identify new Kir6.2-specific compounds

Given the novelty of the identification of a potential drug-binding pocket that could mediate channel opening, with all its implications for K_ATP_ channel pharmacology and therapeutic possibilities, we conducted a virtual library screen (Cardozo and Abagyan, 2005) of the Chembridge Core chemical library of 492,000 drug-like compounds for their biophysical fit to this pocket, as it appears in each of the 3D models of Kir6.2 we obtained, both with and without PIP_2_ present at the location it appears in the PDB 3SYQ structure. The 15 diverse (without common substructures) compounds achieving the threshold docking scores, indicating excellent fit to the shape and electrostatics of the pocket, were selected for *in vitro* testing (Table 1).

### Identification of CL-705G as an opener of K_ATP_ channels

**FIGURE 2:**
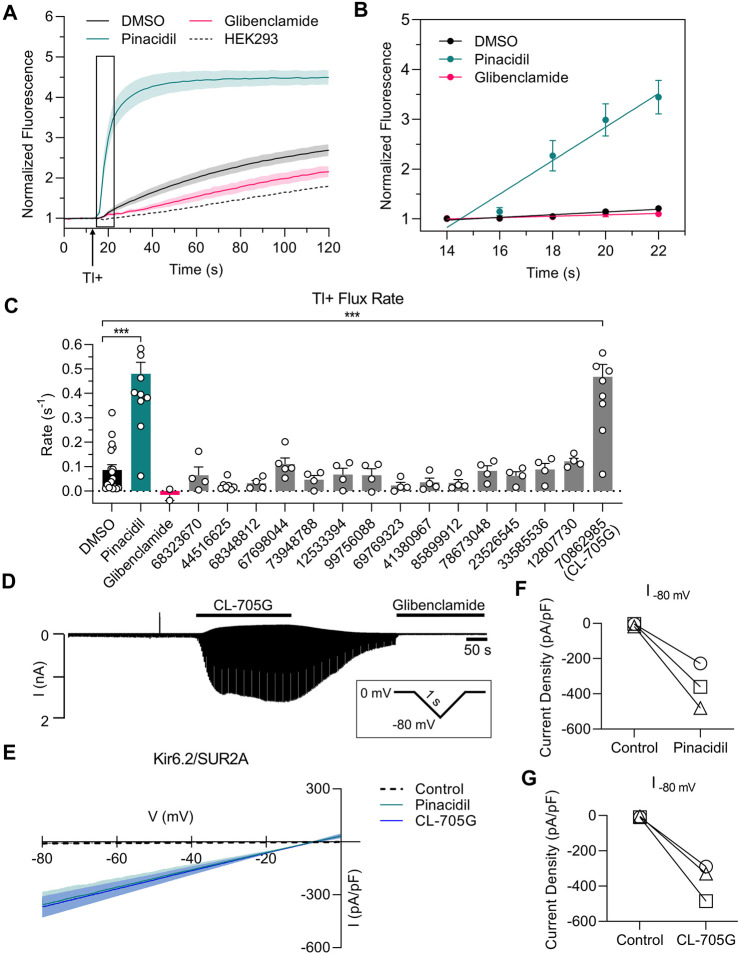
Compound CL-705G is a K_ATP_ channel activator. **(A)** Representative traces depicting Tl^+^ fluorescence measured in Kir6.2/SUR2A-expressing HEK293 cells. Cells were loaded with a Tl^+^ indicator and treated for 10 min with pinacidil (green), glibenclamide (red) or solvent only (DMSO; black). The arrow represents the time at which Tl^+^ uptake was initiated by adding extracellular Tl^+^. The dotted line represents untransfected HEK293 cells. **(B)** The initial Tl^+^ flux rate was calculated by linear regression of the fluorescence data points recorded during the 10–20 s period immediately after adding extracellular Tl^+^ (the boxed area of panel a). **(C)** The initial Tl^+^ flux rate is shown for the different experimental groups. For all groups, flux rate was calculated from technical triplicates for each experiment. Data are summarized from four independent experiments **(D)** Whole-cell voltage clamping was performed using Kir6.2/SUR2A-expressing HEK293 cells. Voltage ramps from 0 to −80 mV were applied every 3 s. Application of CL-705G (100 µM) markedly increased the whole-cell currents, which was reversed upon washout and blocked by glibenclamide (10 µM). **(E)** Current voltage relationships of K_ATP_ channel currents using a 1s ramp pulse from −80 mV to 0 mV. Individual data of current densities recorded at −80 mV before and after **(F)** pinacidil or **(G)** CL-705G. CL-705G. Data are depicted mean ± SEM. ***p < 0.001 using a 1 W ANOVA followed by a Dunnett’s *t*-test.

We initially employed a thallium-sensitive fluorescence assay to assess K_ATP_ channel activity. Experiments were performed with HEK293 cells transiently transfected with Kir6. x/SURx cDNAs, or with HEK293 cell stably expressing Kir6.2/SUR2A or Kir6.1/SUR2B. Cells, grown in 96-well plates, were loaded with a Tl^+^-sensitive dye and Tl^+^ flux was initiated by adding Tl^+^ to the extracellular solution. Addition of Tl^+^ led to a large increase of fluorescence in cells expressing Kir6.2/SUR2A when compared to untransfected cells (Figure 2A). The Tl^+^ flux rate was strongly increased by pinacidil, and inhibited by glibenclamide, demonstrating that the Tl^+^ flux occurred through K_ATP_ channels. We utilized the initial rate of Tl^+^ flux (Figure 2B) as an index of K_ATP_ channel activity. We next tested the effects of 15 of the top ranking compounds identified as potential Kir6.2 binders by virtual library screening (Table 1). Pinacidil and glibenclamide were included as positive controls, respectively to open and block the K_ATP_ channel. The majority of the compounds had little effect on the Tl^+^ influx rate. By contrast, compound CL-705G strongly activated Tl^+^ flux (Figure 2C).

To confirm that CL-705G activates K_ATP_ channels, we performed whole-cell voltage clamping of HEK293 cells expressing Kir6.2/SUR2A. Ramp voltage pulses were applied between 0 and −80 mV. Application of 100 µM CL-705G led to a rapid and reversible increase in K_ATP_ currents (Figures 2D,E). Moreover, the current was blocked by glibenclamide (10 µM), demonstrating the veracity of the recordings. The K_ATP_ channel activation by 100 µM CL-705G was similar to that induced by pinacidil (Figures 2F,G). These data demonstrate that CL-705G activates Kir6.2/SUR2A K_ATP_ channels.

### Structural analogs of CL-705G

**FIGURE 3:**
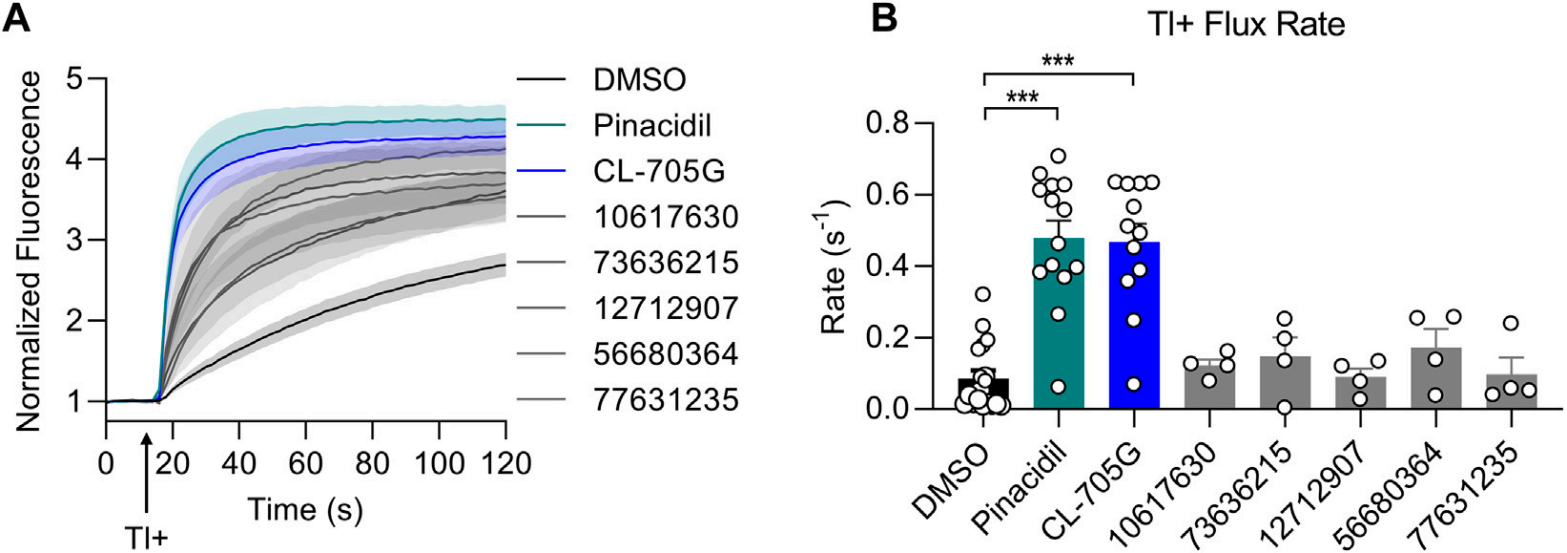
Analogs of CL-705G do not effectively open K_ATP_ channels. Kir6.2/SUR2A expressing cells were treated for 10 min with various compounds (100 µM each) prior to Tl^+^ flux measurements. Depicted are **(A)** fluorescence recordings over time and **(B)** the initial Tl^+^ flux rate. Bar graph values represent the mean ± SEM of at least four experiments, each performed in triplicate. ***p < 0.001 using a 1 W ANOVA followed by a Dunnett’s *t*-test.

We identified nine analogs of compound CL-705G in the Chembridge Core chemical library with 2D structural similarities ranging from 86%–93%. These compounds were docked to our Kir6.2 model as previously described. Of the nine analogs, five docked to the same pocket as CL-705G (Table 2). However, none of the five compounds (100 µM each) resulted in an increased Tl^+^ flux rate comparable to CL-705G or pinacidil (Figure 3). The remainder of the studies therefore focused on CL-705G.

**TABLE 2:** Properties of CL-705G analogs.

ChemBridge Id	Name	Compound structure
10617630	1-(1H-pyrazol-3-ylcarbonyl)-3-{2-[2-(trifluoromethyl)phenyl]ethyl}piperidine	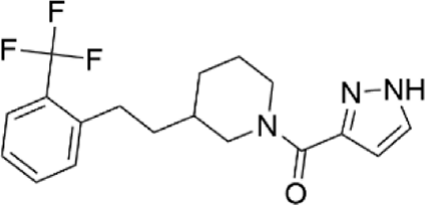
7366215	N-[4,5-dimethyl-2-oxo-3-(trifluoromethyl)-2,3-dihydro-3-furanyl]benzenesulfonamide	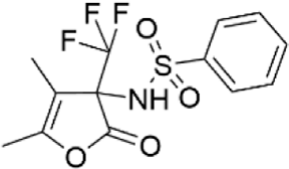
12712907	1-[(4-chloro-1H-pyrazol-3-yl)carbonyl]-3-{2-[2-(trifluoromethyl)phenyl]ethyl}piperidine	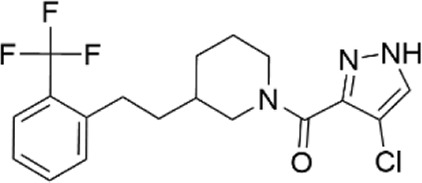
56680364	3-[(3-{2-[2-(trifluoromethyl)phenyl]ethyl}-1-piperidinyl)carbonyl]-1H-pyrazol-5-amine	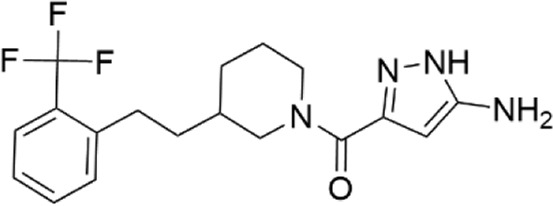
77631235	1-[(1-methyl-1H-pyrazol-5-yl)carbonyl]-3-{2-[2-(trifluoromethyl)phenyl]ethyl}piperidine	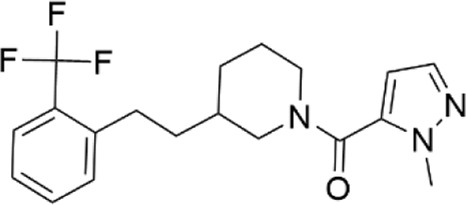

Additional information of each compound is available at Hit2Lead (https://www.hit2lead.com).

### Dose-dependence of CL-705G to activate K_ATP_ channels

**FIGURE 4:**
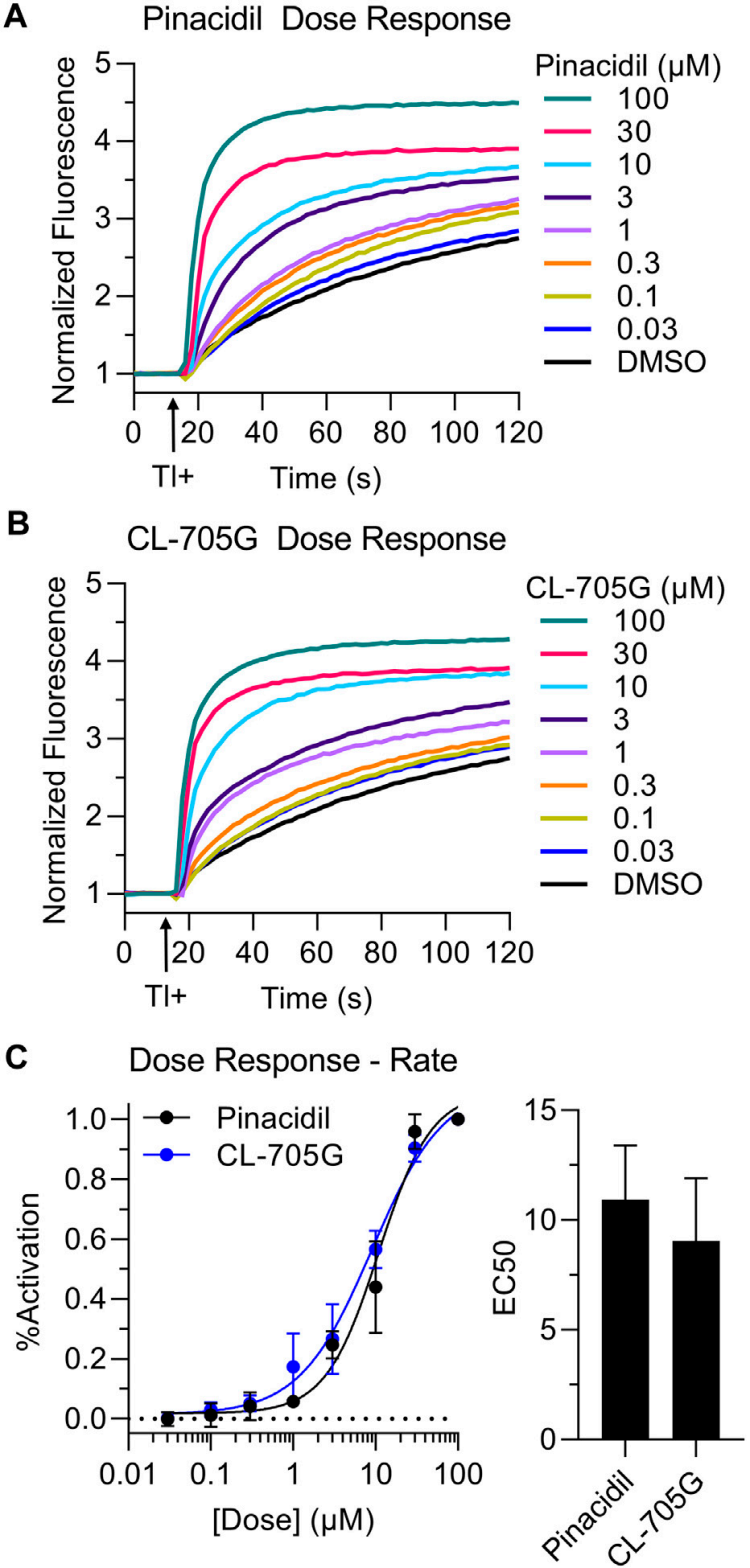
Dose-dependence of CL-705G and pinacidil as K_ATP_ channel openers. The rate of Tl^+^ flux was recorded using Kir6.2/SUR2A-expressing HEK293 cells with various concentrations of **(A)** pinacidil or **(B)** CL-705G. **(C)** The initial Tl^+^ flux rate was calculated at each concentration and normalized to the rate at 100 µM. Data points for each experiment (*n* = 4) were subjected to curve fitting using the following equation (GraphPad Prism): $Y=Y_{Bottom}+\left(X^{HillSlope}\right)*\left(Y_{Top}-Y_{Bottom}\right)/\;(X^{HillSlope}+{EC50}^{HillSlope}$ to obtain the concentration needed for half-maximal activation (EC_50_). The EC_50_ values for the two groups are shown in bar graph format. Data are shown as mean ± SEM (*n* = 4).

Using the Tl^+^ flux assay we next determined dose-response relationships of CL-705G and pinacidil to open K_ATP_ channels. Kir6.2/SUR2A expressing HEK293 cells were preincubated with various concentrations (0.03–100 µM) of CL-705G or pinacidil before determining the initial Tl^+^ flux rates. Similar to published studies (Shindo et al., 1998), pinacidil opened the Kir6.2/SUR2A channel with an EC_50_ of 11 µM (Figure 4). The CL-705G compound had an EC_50_ of 9 μM, demonstrating its potency as a K_ATP_ channel opener similar to pinacidil.

### Specificity of CL-705G as a Kir6.2 opener

**FIGURE 5:**
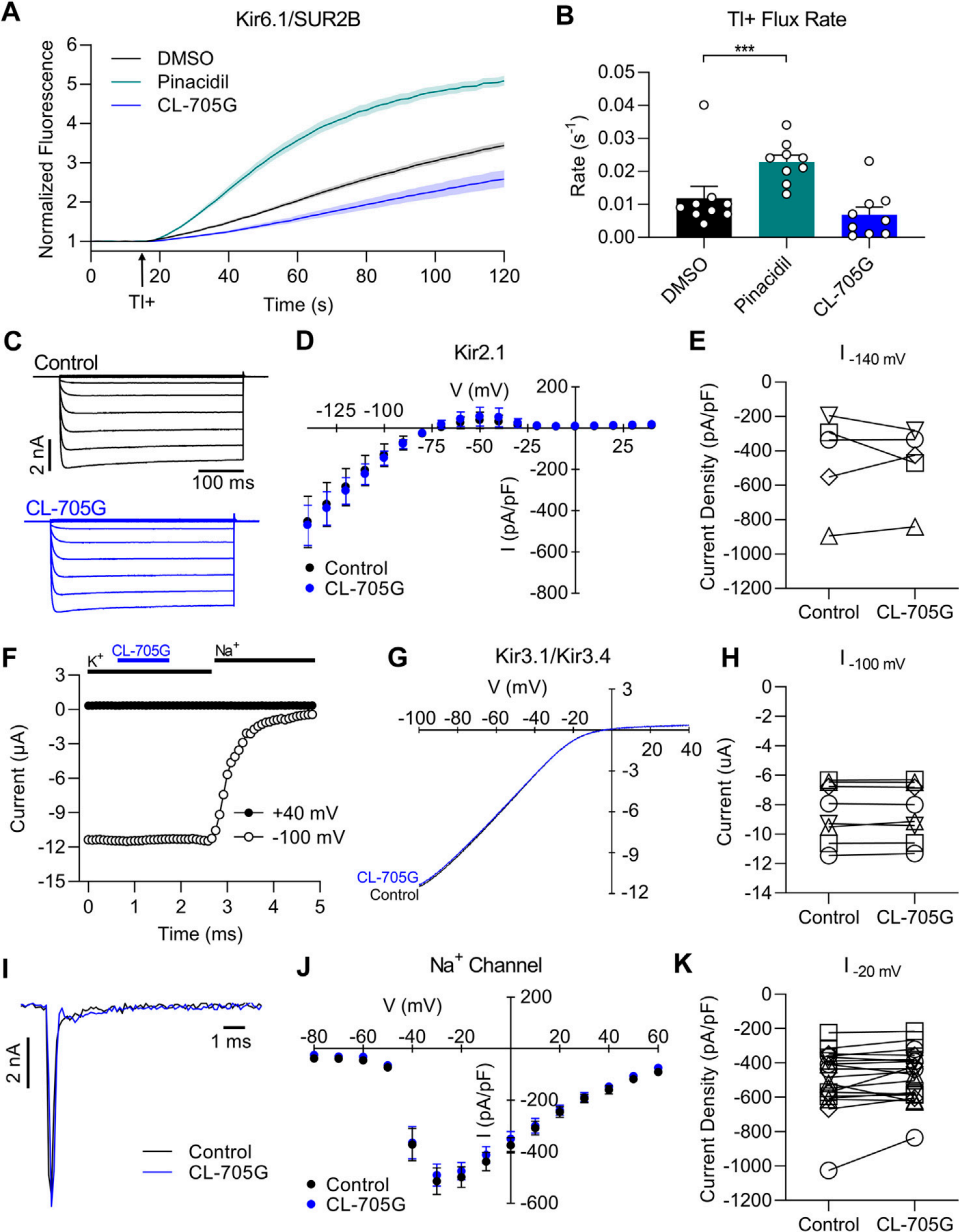
Compound CL-705G does not affect Kir6.1, Kir2.1, Kir3.1/Kir3.4, or Na^+^ channels. **(A)** Tl^+^-induced fluorescence recordings made using Kir6.1/SUR2B-expressing HEK293 cells. Cells were pretreated for 10 min with solvent only (DMSO; black), 100 µM pinacidil (green), or 100 µM CL-705G (blue). **(B)** The initial Tl^+^ flux rate is shown for the three groups (*n* = 9). **(C)** HEK293 cells were transiently transfected with Kir2.1 cDNA and subjected to whole-cell voltage clamping with voltage steps ranging between −140 and 50 mV. Shown are representative raw current traces from a cell before and after application of 100 μM CL-705G. **(D)** Current-voltage relationships were constructed from the Kir2.1 currents densities measured at the end of the voltage step and depicted before and after 5 min of 100 μM CL-705G (*n* = 5). **(E)** The Kir2.1 current densities recorded at −140 mV are shown as individual data points before and after CL-705G. **(F)** Recordings of heterometic Kir3.1/Kir3.4 channels expressed in *Xenopus* oocytes. Shown are representative time course of currents measured at −100 mV (open circle) and +40 mV (closed circle) before and after application of 15 μM CL-705G. The extracellular K^+^ was replaced by 96 mM Na^+^ at the end of the experiment to determine the degree of leak currents. **(G)** Current-voltage relationships of Kir3.1/Kir3.4 currents using a high K^+^ solution and a 1 s ramp pulse from −100 mV to +40 mV repeated every 5 s. Shown is a recording before and after application of 15 μM CL-705G. **(H)** Individual data points of Kir3.1/Kir3.4 currents recorded at −100 mV before and after CL-705G (*n* = 8). **(I)** Whole-cell Na^+^ currents of TE-671 cells were recorded with an automated patch clamp system. Shown are current recordings at 20 mV before (black) after 3 min after application of 100 μM CL-705G (blue). **(J)**. Current-voltage relationships of the Na^+^ current densities are depicted before and after 3 min of 15 μM CL-705G (*n* = 19). **(K)** Individual data of Na^+^ current densities recorded at 20 mV before and after CL-705G. *p < 0.05 vs. control and ***p < 0.001 vs. control, using a one-way ANOVA, followed by Dunnett’s *t*-test.

To determine if compound CL-705G influences the smooth muscle K_ATP_ channel subtype, the Tl^+^ flux assay was repeated using HEK293 cells stably expressing Kir6.1/SUR2B. By contrast to Kir6.2/SUR2A, CL-705G had no effect on Kir6.1/SUR2B at 10 µM (not shown). Even when tested at 100 μM (more than 10x the EC_50_ for Kir6.2), the effect of CL-705G was negligible compared to the vehicle-only control group (Figures 5A,B), despite the high sequence similarity between Kir6.1 and Kir6.2. We further tested each of the 15 identified compounds against Kir6.1/SUR2B channels and found that none of these activated the smooth muscle K_ATP_ channel subtype (Supplementary Figure S1). By contrast, some of these structural homologs, such as 44516625 and 41380967, inhibited TI^+^ flux rates through Kir6.1/SUR2B channels. We chose not to explore this observation further since there are already Kir6.1 blockers available commercially (e.g., PNU-37883 A). Based on these studies, we conclude that CL-705G preferentially open Kir6.2 channels and not Kir6.1 channels. A caveat to these studies is that we have not performed experiments with combinations of Kir6.2/SUR2B, Kir6.1/SUR1 or Kir6.1/SUR2A since these are not thought to occur naturally. Our focus has been to study well-described K_ATP_ subtypes Kir6.2/SUR2A (heart), Kir6.2/SUR1 (pancreas) and Kir6.1/SUR2B (vascular).

We next examined whether compound CL-705G affects other ion channels. We focused on the major Kir channels expressed in the heart to test for cardiotoxicity, including Kir2.1 and Kir3.1/Kir3.4 channels. We conducted whole-cell patch clamping with HEK293 cells transiently transfected with Kir2.1, which produced strongly inward rectifying K^+^ currents. Compound CL-705G (10–100 µM) had no effect on these Kir2.1 current (Figures 5C–E). To determine whether CL-705G affects Kir3 channels, we studied Kir3.1/Kir3.4 heterotetrameric channel heterologously expressed in *Xenopus* oocytyes. At 15 μM, compound CL-705G had no effect on Kir3.1/Kir3.4 currents (Figures 5F–H), and reduced Kir3.1/Kir3.4 currents by 17% at 100 μM, which is 10x higher than the EC_50_ for K_ATP_ channels (data not shown). We also tested the effects of CL-705G against Nav1.7 given its involvement in pain responses (Hameed, 2019). For this experiment, we used cerebellar medulloblastoma TE671 cells, which endogenously express Nav1.7 (Ngum et al., 2022). However, CL-705G had no effect on these Na^+^ channels at 15 μM CL-705G (Figures 5I–K), while a slight inhibition was seen at 100 µM (not shown). Thus, amongst the targets tested, CL-705G preferentially activated Kir6.2-containing K_ATP_ channels. A more comprehensive screen would be needed against other channels before this compound can be considered specific to Kir6.2.

### SUR is required for CL-705G to activate K_ATP_ channels

**FIGURE 6:**
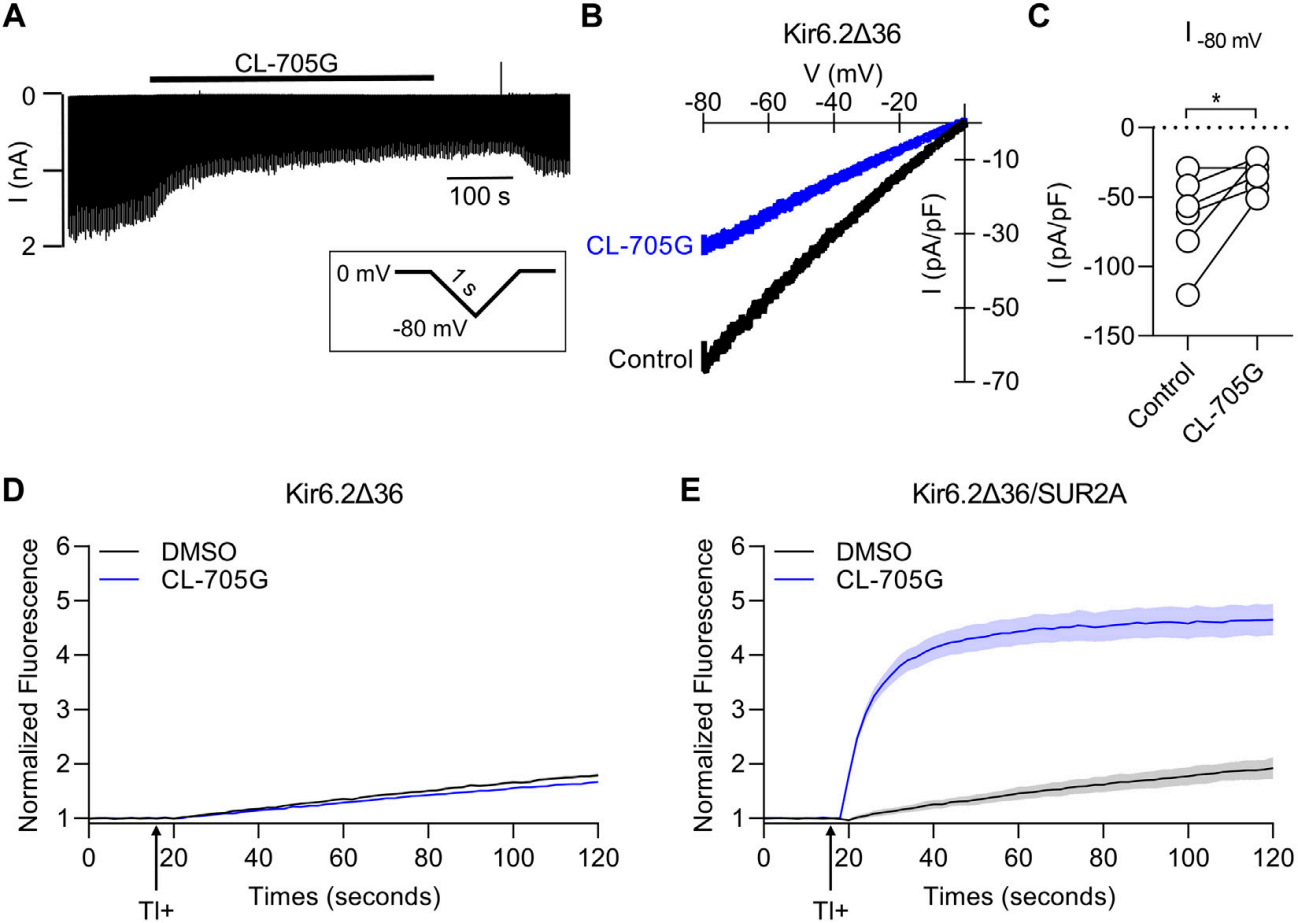
Compound CL-705G requires the presence of SURx to activate Kir6.2-containing channels. HEK293 cells transiently transfected with Kir6.2ΔC36 cDNA in the absence of SUR were subjected to whole-cell voltage clamping using a 2 s voltage ramp to −80 mV from a holding potential of 0 mV. **(A)** A representative recording made before and after the application of 100 μM CL-705G. **(B)** Current-voltage relationships of Kir6.2ΔC36 channel currents using a 1s ramp pulse from −80 mV to 0 mV. **(C)** Individual data points of current densities recorded at −80 mV before and after CL-705G. TL^+^ flux recordings were made using HEK293 cells transiently transfected with **(D)** Kir6.2ΔC36 alone or **(E)** with Kir6.2ΔC36 and SUR2A.

CL-705G can activate Kir6.2 when co-expressed with SUR2A (Figure 2) or with SUR1 (see later). We designed an experiment to determine whether CL-705G can activate Kir6.2 in the absence of a SURx subunit. Normally, only fully assembled Kir6.2/SURx channels effectively traffic to the surface membrane, whereas subunits expressed individually are retained in the endoplasmic reticulum. Removal of the ER retention signal (KRK), for example, by truncation of 36 amino acids of the Kir6.2 C terminus (Kir6.2Δ36), allows a degree of surface expression (Zerangue et al., 1999). We therefore transfected HEK293 cells with Kir6.2Δ36 in the absence of SURx. Whole-cell patch clamping was performed and currents were recorded in response to voltage ramps from 0 to −80 mV. Compound CL-705G did not activate Kir6.2Δ36 currents, but had a slight inhibitory effect, which was reversed upon washout (Figures 6A–C). Similarly, CL-705G had negligible effects on Tl^+^ fluxes of Kir6.2Δ36 when expressed by itself in HEK-293 cells (Figure 6D). By contrast, CL-705G strongly activated Tl^+^ fluxes of Kir6.2Δ36 when co-expressed with SUR2A (Figure 6E). We conclude, therefore, that the SUR subunit is needed for CL-705G to activate Kir6.2-containing K_ATP_ channels.

### Is PIP_2_ required for CL-705G to activate K_ATP_ channels?

**FIGURE 7:**
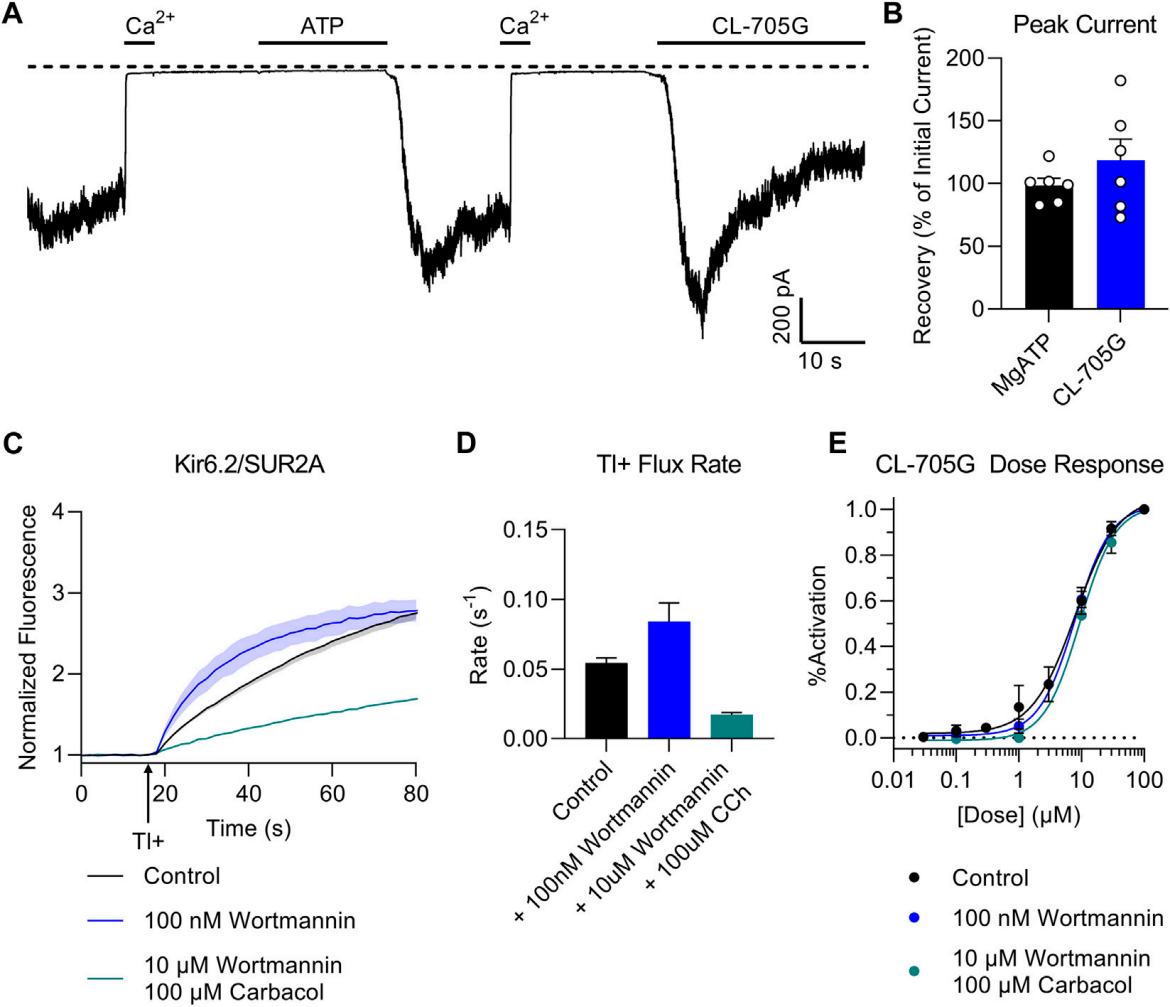
Compound CL-705G activates K_ATP_ channels independent of PIP_2_. **(A)** Inside-out patch clamp recordings were made at a membrane potential of −70 mV using Kir6.2/SUR2A-expressing HEK293 cells. Shown is a representative trace to illustrate Ca^2+^-induced rundown (100 μM Ca^2+^ for 20 s), thought to block the K_ATP_ channel by depleting intracellular PIP_2_. Prolonged exposure to 5 mM MgATP reactivates K_ATP_ channels upon its washout, presumably due to *de novo* PIP_2_ resynthesis. Compound CL-705G (100 μM) activated Kir6.2/SUR2A channels even after a Ca^2+^-induced rundown (PIP_2_-depletion) protocol. **(B)** The degree of activation by CL-705G was calculated relative to the MgATP-dependent activation and summarized as bar graphs (*n* = 6). **(C)** Tl^+^ flux measurements were made using Kir6.2/SUR2A-expressing HEK293 cells pretreated for 60 min with solvent only (control; black), 10 nM wortmannin (blue), or 10 µM wortmannin plus 100 µM carbabcol (blue). **(D)** shown are the initial flux rates of the three groups. **(E)** Using a similar experimental protocol as in the preceding two panels, experiments were performed using Kir6.2/SUR2A-expressing cells pre-treated with various concentrations of CL-705G. Shown are the dose-response curves of CL-705G for the three different groups (*n* = 6). The response (% activation) was calculated after normalizing the data to the maximal responses obtained at 100 µM CL-705G.

Our structural model docks CL-705G in the vicinity of the PIP_2_ binding site (Figure 1). We therefore next examined whether the effect of the compound depends on PIP_2_. In a first approach, we recorded Kir6.2/SUR2A currents in excised membrane patches and employed a method previously described to deplete PIP_2_ at the cytosolic face of the membrane by applying a ∼5s pulse of 100 μM Ca^2+^ (Xie et al., 1999). Consistent with a key role for PIP_2_ in K_ATP_ channel opening (Shyng et al., 2000), this intervention led to a rapid and sustained inhibition of K_ATP_ channel activity (Ca^2+^-induced “rundown”). Prolonged (∼20 s) application of 5 mM ATP activates phosphatidylinositol kinases, leading to *de novo* PIP_2_ synthesis and restoration of PIP_2_ levels (Xie et al., 1999). As a result, K_ATP_ channels are reactivated from Ca^2+^-induced “rundown”, as evidenced by robust K_ATP_ channel current upon ATP washout (Figure 7). Compound CL-705G strongly and rapidly activated K_ATP_ channels even after Ca^2+^-induced “rundown” and PIP_2_ depletion (Figure 7). This result demonstrates that CL-705G can activate K_ATP_ channels in the absence of PIP_2_.

To test whether CL-705G competes with PIP_2_ for a common binding site, we designed an experiment to assess whether interventions that change intracellular PIP_2_ levels would affect the response of the K_ATP_ channel to CL-705G. At nanomolar concentrations, wortmannin specifically blocks phosphoinositide 3-kinases (PI 3-kinases) which degrade PIP_2_, whereas micromolar concentrations also blocks PI 4-kinases, which are required to generate PIP_2_ (Nakanishi et al., 1995). We therefore treated Kir6.2/SUR2A-expressing HEK293 cells with 10 nM wortmannin to elevate PIP_2_ levels (Huang et al., 2011). In another group, we used 10 µM wortmannin, together with 100 µM carbacol to activate muscarinic receptors and phospholipase C, which depletes PIP_2_ (Trebak et al., 2009). Consistent with expectations, low wortmannin concentrations (elevated PIP_2_) led to an increased Tl^+^ flux rate of Kir6.2/SUR2A-expressing HEK293 cells, whereas the higher wortmannin concentration plus carbacol (low PIP_2_) mitigated K_ATP_ channel activity (Figures 7C,D). Neither of these two interventions, however, had any effect on the dose-dependence of CL-705G to activate Kir6.2/SUR2A channels (Figure 7E). This result suggests that the binding sites of CL-705G and PIP_2_ are independent and non-overlapping.

### CL-705G has cardioprotective effects in a cellular model of pharmacological preconditioning

**FIGURE 8:**
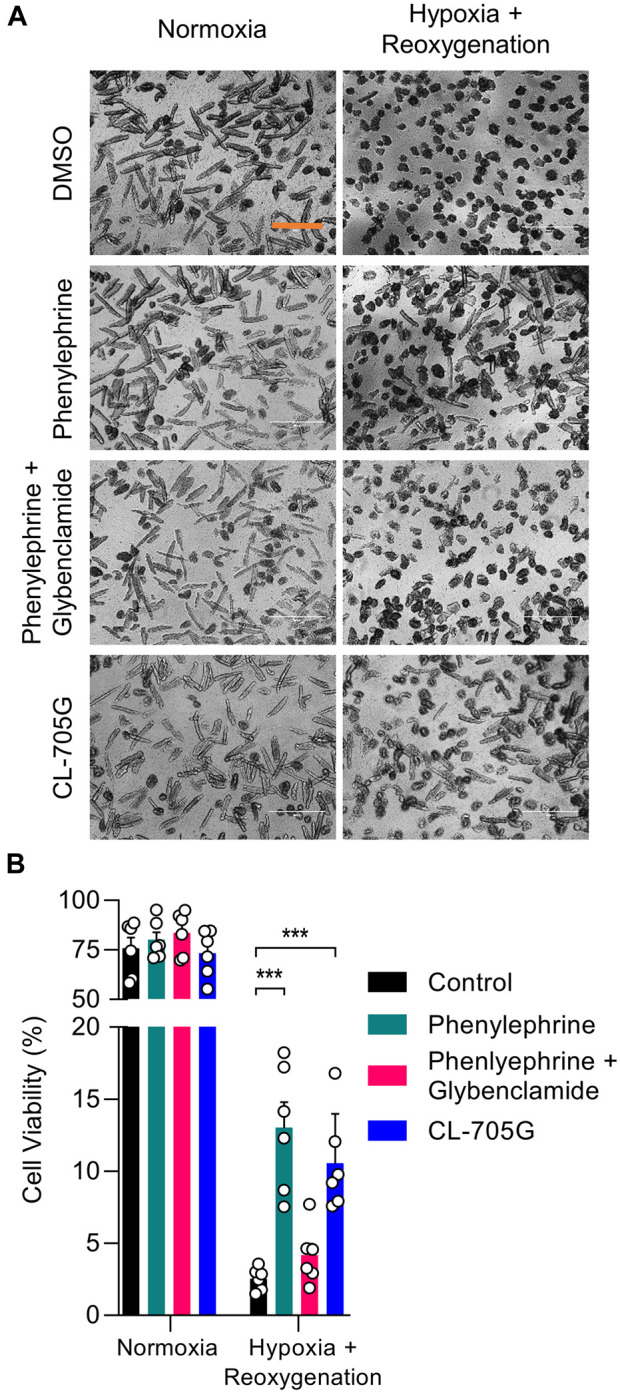
Compound CL-705G has protective properties against hypoxia/reoxygenation injury of cardiomyocytes. Isolated mouse ventricular myocytes were subjected to a hypoxia/reoxygenation assay. Cells were treated with DMSO, phenylephrine (5 μM), phenylephrine with glibenclamide (10 μM), or CL-705G (100 μM) before introducing hypoxia and reoxygenation. **(A)** Representative microscopy images of cardiomyocytes taken after reoxygenation with the various interventions. The scale bar represents 200 μm. **(B)** Cell viability was determined by total cellular ATP content. Summary data are shown for 100,000 cells, determined from 6 measurements made with cells isolated from 2 mice. *p < 0.05 vs. control and ***p < 0.001 vs. control, using a one-way ANOVA, followed by Dunnett’s *t*-test.

Given the cardioprotective effects of K_ATP_ channel opening during hypoxia and ischemic preconditioning, we tested whether CL-705G would have cardioprotective actions. We utilized a model of ischemia and pharmacological preconditioning with isolated mouse cardiac myocytes, in which a protective role of K_ATP_ channels has previously been established (Storey et al., 2013; Yang et al., 2018). In brief, isolated mouse ventricular myocytes were rendered hypoxic by incubation in 1% O_2_ followed by reoxygenation, and cell viability was determined. As expected, pharmacologically preconditioning with phenylephrine improved cell survival in a K_ATP_ dependent manner (Storey et al., 2013). Pre-incubation of the myocytes with 100 µM CL-705G led to a cell survival benefit of the same magnitude (Figure 8). Future experiments can be directed to determine effects of CL-705G on insulin secretion (c.f. diazoxide, to test effects on physiologically relevant Kir6.2/SUR1 in pancreatic β-cells) or to examine effects on blood pressure or blood flow (c.f. pinacidil, to test effects on physiologically relevant Kir6.1/SUR2B in the vasculature).

### Can CL-705G restore the activity of loss-of-function KCNJ11 channelopathies?

**FIGURE 9:**
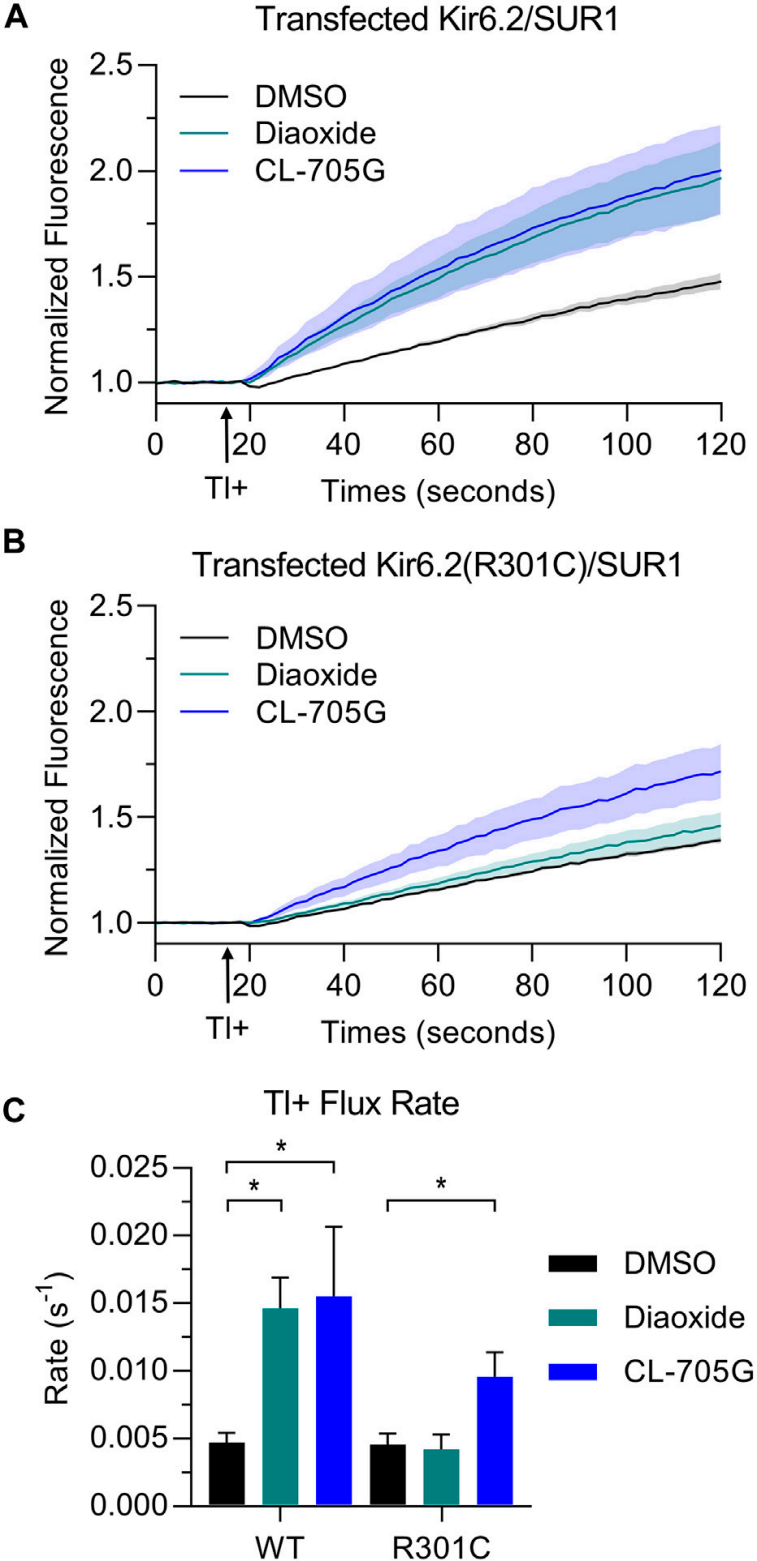
Compound CL-705G increases the Tl^+^ flux rate of a gating-defective Kir6.2-R301C channel. Tl^+^-induced fluorescence recordings were made using HEK293 cells transiently transfected with wild-type Kir6.2 or Kir6.2-R301C, in combination with SUR1 cDNAs. Cells were pretreated for 10 min with solvent only (DMSO; black), 100 µM diazoxide (green), or 100 µM CL-705G (blue). Representative fluorescence traces of **(A)** wild-type Kir6.2/SUR1 or **(B)** Kir6.2-R301C/SUR1 expressing cells. **(C)** Shown are the maximum fluorescence change at the end of the experiment and the initial Tl^+^ flux rate for wild-type Kir6.2/SUR1 (*n* = 4) and Kir6.2-R301C/SUR1 (*n* = 4). Data are shown as mean ± SEM. *p < 0.05 with 1 W-ANOVA followed by Dunnett’s *t*-test compared to DMSO.

Loss-of-function pathogenic variants in *KCNJ11* (the gene that encodes Kir6.2) can lead to congenital hyperinsulinism (CHI), a disorder characterized by persistent β-cell depolarization which results in excessive insulin secretion even in the presence of severe hypoglycemia. We tested whether CL-705G can rescue the gating defects of three such variants using the Tl^+^ flux assay; Kir6.2-F55L, Kir6.2-T72M and Kir6.2-R301C (Lin et al., 2006; Lin et al., 2008). As expected, when co-expressed with SUR1 to recapitulate the pancreatic β-cell K_ATP_ channel, the Tl^+^ flux rates were strongly diminished in all of the variants when compared to wild-type Kir6.2/SUR1. Compound CL-705G did not rescue Tl^+^ flux of Kir6.2-F55L/SUR1 or Kir6.2-T72M/SUR1 (not shown). By contrast, Kir6.2-R301C/SUR1 was minimally activated by diazoxide, but strongly activated by compound CL-705G (Figure 9). We have also tested three loss-of-function gating-defective SUR1 mutants (V715G, S1386P, and L1390P). Based our data, we did not expect CL-705G to activate any of these SUR1 mutants. Indeed, CL-705G did not rescue Tl^+^ flux deficits in any of these SUR1 mutants (data not shown).

## Discussion

We found that compound CL-705G activates Kir6.2/SUR2A channels, demonstrated using a fluorescence-based Tl^+^-flux assay and patch clamping methods. The potency of CL-705G to activate Kir6.2/SUR2A was similar to that of the benchmark K_ATP_ channel activator pinacidil, and it lacked effects on other channels tested, including Kir6.1/SUR2B, Kir2.1, Kir3.1/Kir3.4, and Na^+^ channels in TE671 cells. CL-705G acts similar to PIP_2_ by reactivating K_ATP_ channels after PIP_2_ depletion, but the compound does not appear to compete with PIP_2_ binding. CL-705G had a survival benefit in cardiomyocytes during hypoxia and restored activity of a gating-defective *KCNJ11* missense variant associated with CHI. These findings suggest that CL-705G may be a lead compound to treat some specific K_ATP_ pathologies and channelopathies.

### K_ATP_ channel openers and blockers

Diverse compounds exist that can either inhibit (block) or activate (open) K_ATP_ channel activity (Tinker et al., 2018). Sulfonylureas are a class of K_ATP_ channel blockers and include compounds such as tolbutamide, glibenclamide, glipizide, and gliclazide. Nonsulfonyurea derivatives of glibenclamide (e.g., “glinides”) have emerged as a new generation of K_ATP_ channel blockers, represented by compounds such as meglitinide and repaglinide. Other nonsulfonylurea K_ATP_ channel inhibitors include PNU99963 and PNU37883A. Other compounds with very different structures activate K_ATP_ channel opening. These include benzothiadiazines (diazoxide), pyrimidine sulfates (minoxidil), pyridyl nitrates (nicorandil), benzopyrans (cromakalim), carbothiamides (aprikalim), and cyanoguanidines (pinacidil).

A therapeutic challenge is to find compounds that demonstrate selectivity against a specific K_ATP_ channel subtype. A K_ATP_ channel opener such as diazoxide is a hypotensive agent, but also promotes hyperglycemia, demonstrating significant cross-reactivity between smooth muscle and pancreatic β-cell K_ATP_ channels (Coetzee, 2013). Nicorandil and cromakalim activate both smooth muscle and cardiac muscle K_ATP_ channels. High affinity K_ATP_ channel blockers such as glibenclamide are being used therapeutically to block pancreatic β-cell K_ATP_ channels, but the tissue specificity is marginal since other K_ATP_ channels are effectively blocked at higher concentrations of the drug. Our data show that compound CL-705G shows a degree of selectivity by targeting only Kir6.2-containing channels, such as those in the cardiac ventricle and pancreatic β-cells.

### The binding site of K_ATP_ channel modulators

The vast majority of K_ATP_ channel activators and blockers act by binding to the SURx subunit. Cryo-EM studies have demonstrated, for example, that glibenclamide binds in a pocket located within the transmembrane (TM) region above NBD1, which is framed by TM helices from TMD1 and TMD2 and further stabilized by a hairpin loop in L0 (Driggers and Shyng, 2023). Two amino acid residues in SUR1 (S1238 and T1242) are responsible for increased binding affinity and explains why glibenclamide blocks SUR1 channels better than SUR2x-containing channels. Other K_ATP_ channel blockers, such as repaglinide and carbamazepine bind to a similar region of the channel, but binding is coordinated by different amino acid residues. K_ATP_ channel openers, including NN414, P1075 and levcromakalim also bind to SUR1 and SUR2 within a transmembrane region, where binding is coordinated by specific and overlapping transmembrane helices (Driggers and Shyng, 2023). An important exception is a compound named PNU37886A, which appears to have significant specificity at low micromolar concentrations to block smooth muscle K_ATP_ channels without affecting K_ATP_ currents in cardiac and skeletal myocytes (Wellman et al., 1999; Kovalev et al., 2004). PNU37886A is one of the few compounds that act as a pore-blocker by interacting with the Kir6.1 subunit, and does not act via SURx. Studies to identify new K_ATP_ channel modulators that are specific to a subclass of K_ATP_ channels are therefore needed.

Our virtual library screen was designed to identify compounds that potentially bind to a pocket identified lined by adjacent Kir6.2 subunits in the heteromeric channel complex (Figure 1). The amino acid residues predicted to interact with Kir6.2 are shown in Supplementary Figure S2. Of the 15 compounds initially tested (Table 1) and the five 2D structural homologs (Table 2), CL-705G was the most potent of these compounds to activate K_ATP_ channels, with a potency akin to pinacidil. Pinacidil binds to SUR, with a higher affinity to SUR2A and SUR2B when compared to SUR1 (Moreau et al., 2000). Compound CL-705G, by contrast, might mediate its K_ATP_ channel opening properties by binding to Kir6.2. The presence of SURx is required, however, since when Kir6.2 is expressed by itself (as a C-terminal truncated subunit Kir6.2Δ36), it was not activated by CL-705G. Co-expression of SUR2A with Kir6.2Δ36, by contrast, led to strong activation of K_ATP_ channel activity by CL-705G. We deem it unlikely that CL-705G mediates its effect by binding to SURx since our docking attempts did not support the possibility that CL-705G binds to a conserved pocket of SUR1 or SUR2 (not shown). Moreover, if CL-705G activated K_ATP_ channels by binding to SUR2, then we would expect Kir6.1/SUR2B channels to be activated at least partially. By contrast, the Kir6.1 channels were unaffected by CL-705G even at 10x the effective concentration needed to activate Kir6.2. A more likely explanation is that the CL-705G binding pocket in Kir6.2 is disrupted in the absence of SURx, or alternatively that SUR participates in CL-705G binding to Kir6.2. The latter possibility is supported by the proximity of SUR Loop 0 to the pocket to which CL-705G docks (not shown).

### The mechanism by which CL-705G activates K_ATP_ channels

Since CL-705G is predicted to bind in close proximity of the PIP_2_ binding site (Figure 1), we tested whether they interact. Following Ca^2+^-induced rundown, which inactivates K_ATP_ channels by depleting PIP_2_ levels (Xie et al., 1999), CL-705G led to a rapid and robust K_ATP_ channel activation. This result might be interpreted that CL-705G rescues the channel from an inactivated state. Certain Kir6.2 mutations inhibit gating by promoting an inactivated state, from which channel activity can be rescued by ATP (in the absence of Mg^2+^) or by the non-hydrolysable AMP-PNP (Lin et al., 2008). Indeed, the finding that the compound rescued the activity of one of these gating-defective Kir6.2 mutants (Kir6.2-R301C) is consistent with this interpretation. If so, however, it would not be clear why CL-705G specifically rescued R301C, and no other similar Kir6.2 mutants that enter the inactivated state (F55L and T72M) or any of the SUR1 mutants tested (V715G, S1386P, and L1390P). Moreover, there is little evidence that wild-type Kir6.2 channels enter the inactivated state and the data in Figure 9 cannot easily be explained by this mechanism. Another possibility is that CL-705G interacts with, or substitutes for, PIP_2_ to activate the K_ATP_ channel. We tested this possibility by determining whether PIP_2_ changes the EC_50_ of CL-705G. Even though our interventions to manipulate PIP_2_ (low vs. high wortmannin concentrations) had the expected effects in baseline K_ATP_ channel activity, we not observe an obvious change to the apparent EC_50_ of CL-705G, suggesting that PIP_2_ and CL-705G bind to distinct sites. A possible mechanism is presented in Supplementary Figure S2, which demonstrates that CL-705G may bind to residues in the M2 transmembrane helix, as well as residues in the Kir6.2 C-terminus. Thus, CL-705G may bridge these functional domains to cause a conformational change that favors the channel open state. Some of these amino acid residues are conserved in Kir6.1. However, the Kir6.1 C-terminus is offset from the membrane interface by ∼5.8 Å relative to Kir6.2 (Sung et al., 2021), which would preclude CL-705G from having a similar effect, which may partially explain the specificity of the compound against Kir6.2. Further studies are needed to test these ideas.

## Conclusion

Compound CL-705G activates Kir6.2-containing K_ATP_ channels without affecting structurally similar Kir channels such as Kir6.1/SUR2B, Kir2.1, or Kir3.1/Kir3.4. Compound CL-705G is a possible lead compound to K_ATP_ channel related pathologies (such as cardiac ischemia) or to treat specific *KCNJ11* missense channelopathy loss-of-function variants.

## Data availability statement

The original contributions presented in the study are included in the article/Supplementary Material, further inquiries can be directed to the corresponding author.
