# Supplementary material for: CL-705G: a novel chemical Kir6.2-specific KATP channel opener
Source: Front Pharmacol. 2023 Jun 20;14:1197257. doi: 10.3389/fphar.2023.1197257 (PMC10319115; doi:10.3389/fphar.2023.1197257)
Supplement: Supplementary file 1 [file Table1.DOCX]

**Supplemental Figures**


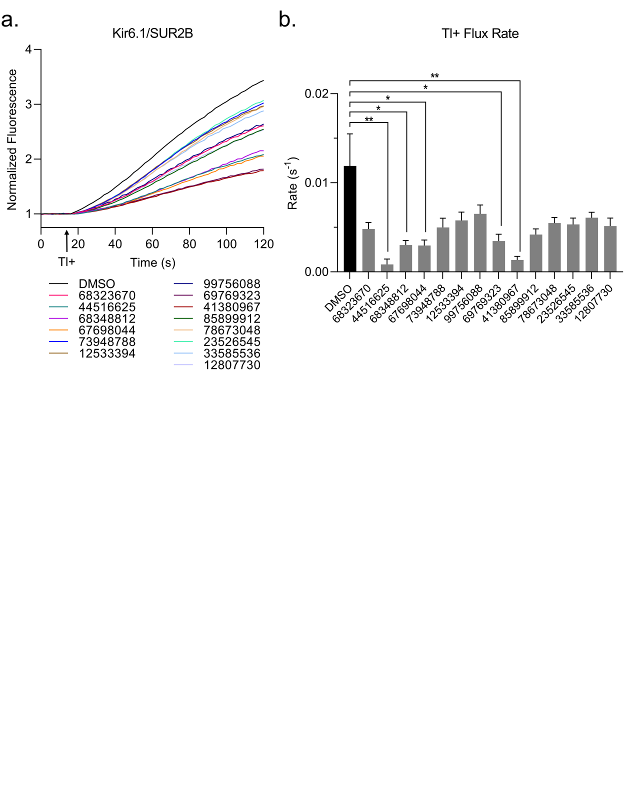


**Figure S1**: None of the compounds activated Kir6.1 currents. (a) Representative traces depicting Tl^+^ fluorescence measured in Kir6.1/SUR2B-expressing HEK293 cells. Cells were loaded with a Tl^+^ indicator and treated for 10 min with the various compounds (100 µM each) or with solvent-only as negative control (DMSO). The arrow represents the time at which Tl^+^ uptake was initiated by adding extracellular Tl^+^. (b) The initial Tl^+^ flux rate was calculated by linear regression of the fluorescence data points recorded during the 10-20 s period immediately after adding extracellular Tl^+^. Data are depicted mean ± SEM. *p<0.05, **p<0.01 and ***p<0.001 using a 1W ANOVA followed by a Dunnet’s t-test.


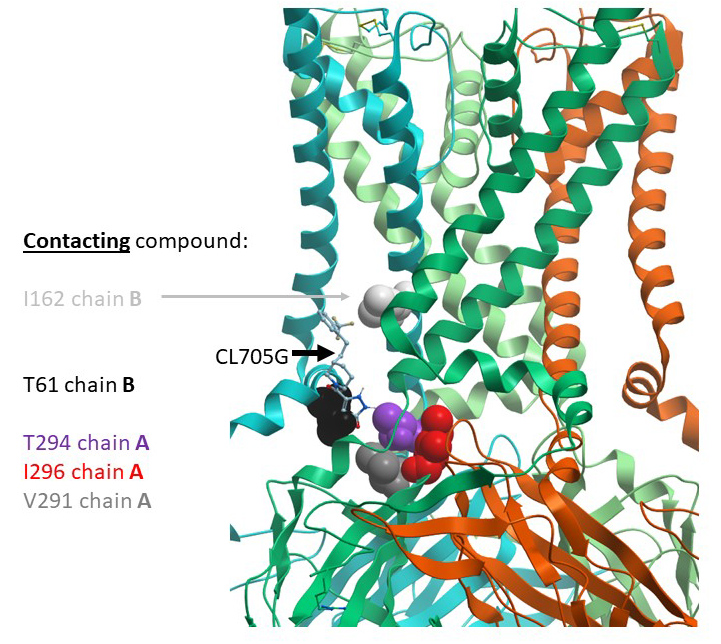


**Figure S2**: Amino acids forming the CL-705G pocket are labeled and color coded according to the legend. The backbone of Kir6.2 is depicted as a ribbon, with each monomer colored differently. I162 from one Kir6.2 monomer forms the "roof" of the pocket in this perspective and V291/T294/I296 from an adjacent monomer form the "floor".
